# Assessing Multi-Attribute Characterization of Enveloped and Non-Enveloped Viral Particles by Capillary Electrophoresis

**DOI:** 10.3390/v14112539

**Published:** 2022-11-17

**Authors:** Rita P. Fernandes, José M. Escandell, Ana C. L. Guerreiro, Filipa Moura, Tiago Q. Faria, Sofia B. Carvalho, Ricardo J. S. Silva, Patrícia Gomes-Alves, Cristina Peixoto

**Affiliations:** 1 iBET, Instituto de Biologia Experimental e Tecnológica, Apartado 12, 2780-901 Oeiras, Portugal; 2 Instituto de Tecnologia Química e Biológica António Xavier, Universidade Nova de Lisboa, Avenida da República, 2780-157 Oeiras, Portugal

**Keywords:** bioanalytical tools, capillary electrophoresis, quality attributes, virus-based therapeutics

## Abstract

Virus-based biopharmaceutical products are used in clinical applications such as vaccines, gene therapy, and immunotherapy. However, their manufacturing remains a challenge, hampered by the lack of appropriate analytical tools for purification monitoring or characterization of the final product. This paper describes the implementation of a highly sensitive method, capillary electrophoresis (CE)-sodium dodecyl sulfate (SDS) combined with a laser-induced fluorescence (LIF) detector to monitor the impact of various bioprocess steps on the quality of different viral vectors. The fluorescence labelling procedure uses the (3-(2-furoyl) quinoline-2-carboxaldehyde dye, and the CE-SDS LIF method enables the evaluation of in-process besides final product samples. This method outperforms other analytical methods, such as SDS–polyacrylamide gel electrophoresis with Sypro Ruby staining, in terms of sensitivity, resolution, and high-throughput capability. Notably, this CE-SDS LIF method was also successfully implemented to characterize enveloped viruses such as Maraba virus and lentivirus, whose development as biopharmaceuticals is now restricted by the lack of suitable analytical tools. This method was also qualified for quantification of rAAV2 according to the International Council for Harmonisation guidelines. Overall, our work shows that CE-SDS LIF is a precise and sensitive analytical platform for in-process sample analysis and quantification of different virus-based targets, with a great potential for application in biomanufacturing.

## 1. Introduction

Virus-based biopharmaceuticals have a proved potential in different clinical applications, but, due to their complexity, their bioprocessing represents an essential and challenging task nowadays. In fact, regulatory requirements have been evolving and becoming more challenging during the last decades, reflecting the increasing complexity of biopharmaceutical products [1]. Virus structures can undergo several modifications during bioprocessing, temperature, ionic strength, pH, or shear can compromise viral vector functionality or infectivity [1,2]. Considering this, a set of complementary analytical tools needs to be in place to assess the identity, quantity, potency, and purity of the virus-based targets to monitor the bioprocess and guarantee the safety and efficacy of the final drug product. Analytical methods currently available for this purpose have limited sensitivity and robustness, thus limiting their general applicability, especially for in-process samples [3,4]. In the end, besides accurate analytical tools that ensure compliance with the regulatory guidelines, there is also a need for fast and robust techniques to accelerate their translation from development to market [5].

Capillary electrophoresis (CE) has undergone a wide re-emerging in the analytical field since the pharmaceutical industry moved from traditional small therapeutics into complex biologicals, such as protein- and virus-based products [6]. This methodology enables the separation through different modes, including capillary isoelectric focusing (cIEF), gel-based CE-sodium dodecyl sulfate (SDS), and capillary zone electrophoresis (CZE), providing a comprehensive characterization of the bioproduct. In addition, recent developments in the capillary material used for the separation of DNA fragments [7], show that there is still potential to improve this technology for biopharmaceutical applications. CE has already proved to be a powerful orthogonal technique for several bioanalytical applications, such as purity and structural protein confirmation [8], analyte identification [9], molecular interactions [10], and immunoaffinity assessment [11]. Moreover, CE can be used on-line with other analytical techniques, such as high-resolution mass spectrometry, scaling its analytical potential [11,12]. CE-SDS is currently widely used in the pharmaceutical industry to assess sample purity of therapeutic proteins, particularly monoclonal antibodies (mAbs) by ultraviolet (UV) light detection. However, this type of detection requires a large amount of product, making it unsuitable for virus-based pharmaceuticals, where total protein concentration is considerably lower [13]. The use of a laser-induced fluorescence (LIF) detector is an established alternative to reduce the sample amount, offering additional advantages, such as high resolution and improved sensitivity [14]. For this type of detector, the sample needs to be labelled with either non- or covalently bound fluorescent dyes to be detected [15]. For the case of mAbs, CE-SDS with LIF detection of 3-(2-Furoyl)quinoline-2-Carboxaldehyde (FQ)-labelled samples has already been validated as a GMP method for purity assessment [16,17]. In addition, the CE-SDS LIF methodology with pyrylium dye-labelled samples is also being explored for purity analysis and quality control of recombinant adeno-associated virus (rAAV) capsid proteins [18].

Here, we implemented a robust and sensitive analytical platform for in-process sample analysis and quantification for both non- and enveloped viral vectors using FQ labelling, allowing the detection of viral proteins through CE-SDS coupled to a LIF detector. The established method was used to assess one of the most important critical quality attributes (CQA) of rAAV, the ratio of two structural viral proteins (VP), VP1 and VP3, using 100 times less sample than traditional methods (e.g., SDS-PAGE). In addition, we analyzed several samples from purification processes of either non-enveloped rAAV or enveloped virus, lentivirus (LV), recombinant vesicular stomatitis virus (rVSV—Maraba MG1 vector), and a chimeric VSV with proteins of Newcastle Disease Virus (rVSV-NDV). The increased sensitivity of the method allowed a better understanding of virus quality, namely, purity and integrity. Moreover, qualification of the rAAV quantification method using a commercial reference standard was pursued following the International Council for Harmonisation (ICH) guidelines [19]. Altogether, this work demonstrates the high sensitivity and versatility of this methodology that may be applied for the characterization of either enveloped or non-enveloped viruses-based products and can contribute to bridge a critical gap in the bioanalytical field and virus particles biomanufacturing.

## 2. Materials and Methods

### 2.1. Purified Samples

Both non-enveloped, such as rAAV, and enveloped viruses, such as LV, rVSV–Maraba MG1 vector, and a chimeric rVSV-NDV, were evaluated in this work. A quality screen of different rAAV serotypes from different production platforms (already described and published) was also conducted. rAAV2 was produced in HeLaS3 stable cell line system [20]. rAAV5, rAAV8, and rAAV9 were produced by triple plasmid transfection methodology by suspension cultures of HEK293T, as described in Chahal et al., 2014 [21], with the following modifications: culture medium used was BalanCD HEK293 (FUJIFILM, Tokyo, Japan) supplemented with 4 mM GlutaMAX(TM) (Thermo Fisher Scientific, Waltham, MA, USA). Transfection was performed at a cell density of 2 × 10^6^ cell/mL and harvest was performed 72 h post transfection. LVs were produced with HEK293T cells by the four-plasmid transfection system, consisting of three structural plasmids and one plasmid corresponding to the transgene, which, in this case, was GFP [22]. The rVSV was kindly provided by Turnstone Biologics and the chimeric rVSV-NDV vector was kindly donated by Dr. Jennifer Altomonte from Technical University of Munich.

All the samples were stored at −80 °C and thawed immediately before sample preparation for CE-SDS LIF analysis.

### 2.2. In-Process Samples

For in-process virus-based samples analysis on CE-SDS LIF, two different purification processes for LV and rAAV were used. LV downstream purification process was based on the protocol described previously by Moreira et al. [23]. Samples from the two elution fractions (high and low ionic strength step) were evaluated by CE-SDS LIF in terms of purity and LV particle integrity.

For the rAAV2, the following downstream processing steps were performed: cell lysate was clarified by centrifugation (4000× *g* for 5 min at 20 °C) and filtration with a polyethersulfone (PES) membrane 0.2 µm filter (Supor^®^, Pall Life Sciences, Portsmouth, UK), followed by an affinity chromatography step with a AVB Sepharose column (Cytiva, Marlborough, MA, USA) [24]. The polishing step for removal of residual impurities consisted of size exclusion chromatography (SEC) with Sepharose 4 Fast Flow resin (Cytiva). Samples from AVB and SEC steps were evaluated by CE-SDS LIF in terms of purity and the presence of process-related impurities.

### 2.3. Sample Preparation for CE-SDS LIF 

The sample labelling procedure for CE-SDS LIF analysis was based on the protocol described by SCIEX (Framingham, MA, USA) [25]. For the purity evaluation analysis, 10 µL of each virus sample was mixed with 2 µL of an aqueous solution with 4% SDS (Life-Technologies) and 150 mM N-Ethylmaleimide (NEM) (Sigma-Aldrich, Burlington, MA, USA) and incubated at 70 °C for 5 min, for denaturation. After that, the ATTO-TAG™ FQ Amine-Derivatization Kit (Life Technologies, Carlsbad, CA, USA) was used to label the samples. A volume of 2.5 µL of FQ dye at a concentration of 2.5 µM and 1 µL of the nucleophile KCN solution (30 mM) were added. After incubation at 70 °C for 10 min, 28 µL of an aqueous solution of 1% SDS was added to quench the labelling reaction, followed by another incubation for 5 min. Before injecting the sample into the CE LIF instrument, a dilution was performed with 40 µL of Liquid Chromatography-Mass Spectrometry (LC-MS) grade water (Fisher Scientific). To achieve higher resolution and sensitivity, the sample was desalted before being loaded into the analysis vials. This desalting step employed a 10 kDa cut-off filtration membrane (Amicon^®^ Ultra—10 kDa 0.5 mL centrifugal filters). The buffer exchange solution was composed of an aqueous solution of 0.1% SDS and 1.875 mM NEM. For this, two consecutive centrifugations of 10 min at 20,817× *g* were performed.

### 2.4. CE-SDS LIF Instrument Setup

All the analyses were carried out in a CESI 8000 Plus system (SCIEX) with a 488 nm laser-induced fluorescence (LIF) detector module. Detection was performed using an emission filter of 600 nm. The separation was performed using a bare fused silica capillary of 50 µm inner diameter (I.D.), 30.2 cm of total length, and 20.2 cm of effective separation length (SCIEX).

The overall conditioning, separation, and shutdown methods’ settings for the time program was performed as depicted in Table 1 [26].

Conditioning and shutdown methods were run at the beginning and end of every analysis sequence, respectively.

For the separation of viral proteins, a standardized method was designed. The samples were injected by applying a voltage of 5 kV for 20 s. The separation was carried out either for 25 or 30 min with a voltage of 15 kV, depending on the type of sample (25 for AAVs, and 30 min for the analyzed enveloped targets).

### 2.5. CE-SDS LIF Qualification as a Quantification Method for AAV

A commercially available reference standard of rAAV2 was used (ATCC, ref: VR-1616) for method implementation and qualification of the quantification method. Specificity for the AAV2 viral proteins, absence of matrix interference and peak assignments were established during method development, by using blanks constituted of sample matrix. The method repeatability and linearity were determined based on 6 concentration levels in the range of 1.15 × 10^10^ TP–3.67 × 10^11^ TP rAAV2 particles per mL (standard curve). For the qualification, an experimental sequence was designed with 3 replicates of the standard curve, plus 3 concentration levels of quality control (QC) samples—low, middle, and high range with 1.84 × 10^10^, 3.67 × 10^10^, and 1.47 × 10^11^ TP/mL, respectively. A 10 kDa marker (SCIEX) was included in each concentration point of the calibration curve as an internal standard. The labelling of this internal standard was performed separately, following the samples preparation herein presented, and afterwards 1 µL of this preparation was added to each concentration sample. 

This experimental sequence enabled the assessment of linearity, range, accuracy, repeatability, intermediate precision, limit of detection (LOD), and limit of quantification (LOQ). For this, the corrected peak areas ($A_{corr}$) of VP3 and 10 kDa internal control peaks were calculated, using the velocity correction as shown in Equation (1). Here, we used the uncorrected peak areas (A), and the migration times (*t*) and capillary effective length ($L_{d}\;$)(to calculate the velocity (ν)).

(1) $$A_{corr}=\nu A=\frac{L_{d}A}{t}$$

### 2.6. CE-SDS UV 

For the electrophoretic separation using the UV light detector, 100 µL of sample was added to a 10 kDa Amicon tube (Amicon^®^ Ultra—0.5 mL centrifugal filters) and desalted in two rounds with sample buffer (SCIEX). After desalting, 2 µL of 10 kDa marker (SCIEX), 3 µL of 1 M Dithiothreitol (DTT) and 10 µL of SDS 1% were added, followed by a 10 min incubation at 100 °C. After denaturation and reduction, 45 µL of LC-MS water was added to the mix and transferred to the nanoVial for further analysis on the CESI Plus 8000 system. The experimental conditions used for the conditioning, shutdown and separation methods were performed as for CE-SDS LIF method, changing only the detector for UV light (214 nm).

### 2.7. SDS-PAGE with Sypro Ruby Pro Staining

For protein profiling analysis in traditional SDS-PAGE, 13 µL of each sample was mixed with the loading buffer (LDS sample buffer 4× (Thermo Fischer Scientific), and sample reducing agent 10× (Invitrogen, ref. NP004, Waltham, MA, USA) and denaturized at 99 °C, for 5 min. MG1 proteins were separated under reducing conditions in a 4–12% (*w*/*v*) polyacrylamide NuPAGE^®^ gradient precast gel (Thermo Fischer Scientific). Samples were resolved for 60 min using NuPAGE^®^ MOPS SDS running buffer at a constant voltage of 200 V and stained with Sypro ruby pro (Thermo Fischer Scientific) overnight following the manufacturer’s protocol.

### 2.8. Quantification of Adenovirus 5 by qPCR

The quantification of wild-type Adenovirus 5 (wtAd5) was performed by qPCR as described previously by Martin et al. [20], with minor modifications. Briefly, in-process samples were treated with DNAse (Promega, Madison, WI, USA) followed by Proteinase K (Roche, Basel, Switzerland) treatment and a heat inactivation step 20 min at 95 °C. wtAd5 viral genome was detected with specific primers (Forward primer 5′-TCCGGTTTCTATGCCAAACCT-3′, reverse primer 5′-TCCTCCGGTGATAATGACAAGA-3′) [27] against E1A gene and quantified by standard curve extrapolation approach.

### 2.9. Quantification of Adeno-Associated Virus by ELISA

The quantitative determination of AAV serotype 2, 5, 8, and 9 particles was performed through enzyme immunoassay using the respective titration enzyme-linked immunoassay (ELISA, Helsinki, Finland) kit (Progen, Heidelberg, Germany), following the manufacturer’s protocol. This method is based on the sandwich ELISA technique. For particle concentration, three separate dilutions of each sample were performed. After the assay, the absorbance was measured at 450 nm and 650 nm for reference value, on Infinite^®^200 PRO NanoQuant (Tecan, Mennedorf, Switzerland) microplate multimode reader.

### 2.10. Lentivirus Infectivity Assay

The functional LV titers were determined by transducing HEK293T cells with the produced LV supernatants. GFP expression was analyzed by flow cytometry as described elsewhere [22].

### 2.11. Lentivirus Total Particles Quantification

Total particles (TP) concentration was calculated by determining the amount of p24 protein using the QuickTiter™ Lentivirus Titer Kit (Cell Biolabs, Inc., San Diego, CA, USA) according to the manufacturer instructions. A ratio of 1.25 × 10^7^ TP/ng of p24 was used to calculate the TP titer.

## 3. Results

### 3.1. Capillary Gel Electrophoresis Analysis of Virus-Based Products

**Figure 1:**
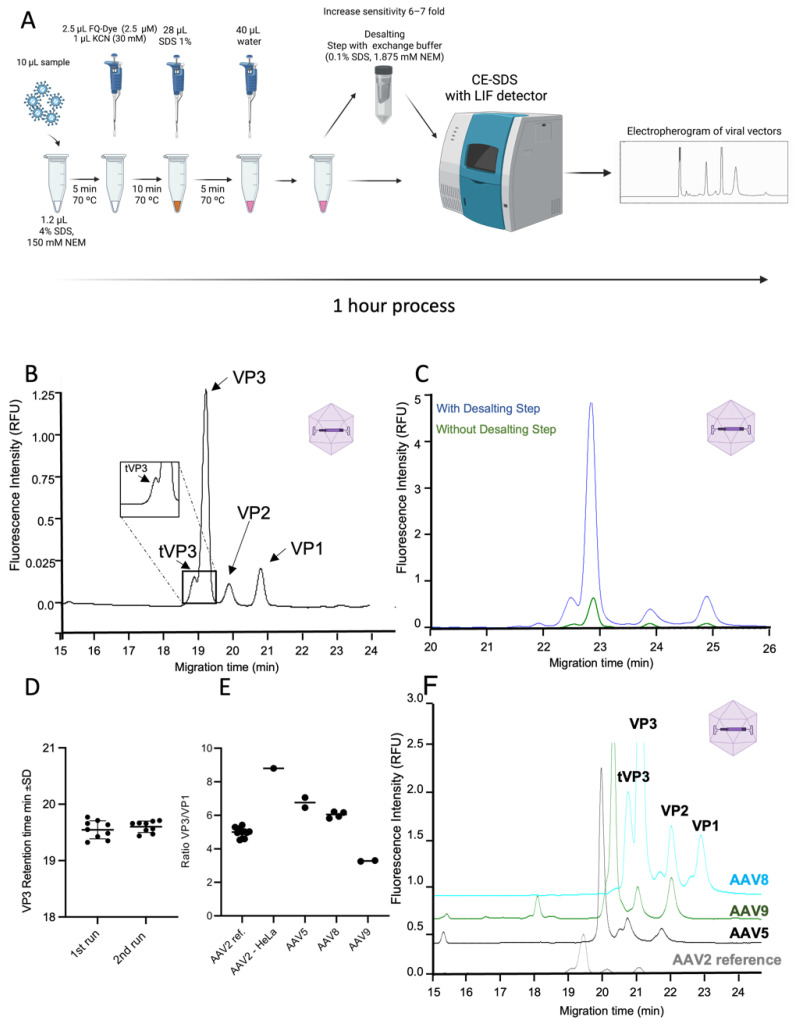
CE-SDS for the detection of AAV viral capsid proteins. (**A**) Representation of the methodology used for the analysis of viral proteins using CE-SDS coupled with a LIF detector. (**B**) Electropherogram of rAAV2 reference standard, displaying capsid proteins VP1, VP2, VP3, and tVP3. (**C**) Comparison of rAAV2 (9 × 10^11^ TP/mL) electropherograms using desalting step (blue line) vs no desalting (green line). (**D**) Representation of VP3 migration time (min) for two different sample preparations (*n* = 9 for each). (**E**) Comparison of CQA attribute (ratio VP3/VP1) for different AAV serotypes. (**F**) Overlay of the electropherograms of the different AAV serotypes; (HeLa3 AAV2 9 × 10^12^ viral genomes (VG/mL), with rAAV5 (2 × 10^11^ VG/mL), rAAV8 (5 × 10^12^ VG/mL), and rAAV9 (7 × 10^12^ VG/mL).

To evaluate the applicability of the CE-SDS LIF method for different viral vectors, we first used the commercial reference standard rAAV2. Several amounts of rAAV2, ranging from 4.9 × 10^8^ to 1.5 × 10^7^ TP, were labelled with FQ dye and injected into a CESI 8000 Plus instrument (Figure 1A). The AAV capsid is composed of 60 copies in total of viral protein VP1 (∼87 kDa), VP2 (∼73 kDa), and VP3 (∼61 kDa) in an approximate population ratio of 1:1:10, respectively [28]. The electropherogram of a representative single injection of the rAAV2 sample is depicted in Figure 1B, showing a successful separation of all three viral proteins of rAAV2 (VP1, VP2 and VP3), achieved within 22 min of run time. The viral proteins were detected with baseline separation, at an average migration time (MT) of 21.05, 20.11, and 19.42 min, respectively. CE-SDS LIF methodology allowed us to detect a peak corresponding to a truncated form of VP3 (tVP3), which is known to be present in serotype 2, at 19.09 min MT [18]. We also evaluated the influence of a desalting step using a centrifugal filtering device (Amicon™ with a pore size of 10 KDa) with the same amount of sample injected, as presented in Figure 1C. We observed an increased sensitivity by approximately 6–7-fold when compared with no desalting. This result supports prior reports detailing how high salt concentrations in the injected sample reduce CE-SDS sensitivity [29]. We estimate that the FQ labelling/LIF detector approach described here increases sensitivity by 100-fold when compared to previous reports using CE-SDS with a UV detector [29]. Moreover, the performance consistency of the equipment was evaluated through two independent experiments, including different sample preparations with several injections each (Figure 1D). The analysis of the migration time of the VP3 protein showed an average value of 19.57 min, with a relative standard deviation (RSD) of 0.67% (*n* = 9), showing great repeatability.

The CE-SDS LIF methodology was also used for the evaluation of VP3/VP1 ratio, a known CQA for the rAAV product. We analyzed the serotypes rAAV2, rAAV5, rAAV8, and rAAV9, by quantifying corrected peak areas of the VP3 and VP1. The results of VP3/VP1 ratios obtained by CE-SDS LIF showed excellent consistency over all serotypes as shown in Figure 1E,F. Interestingly, by using a CE-SDS LIF analytical approach, we were able to detect significant differences in rAAV2 viral vector quality among two production platforms evaluated. While rAAV2 produced in HEK293T by triple plasmid transfection (reference material) had a VP3/VP1 ratio of 5.6, the HeLa S3-based production system showed a VP3/VP1 ratio with a value of 9, near to the theoretical optimal VP composition of the AAV capsid of 10 [30]. These results suggest that CE-SDS LIF method is capable of detecting differences associated with other quality criteria, besides assessing purity. Notably, when compared to existing methods for assessment of AAV capsid protein purity, such as SDS-PAGE [31], FQ-labelled protein analysis on the CE-SDS LIF platform reduces analysis time while increasing sensitivity, and precision of VPs ratio measurement.

**Figure 2:**
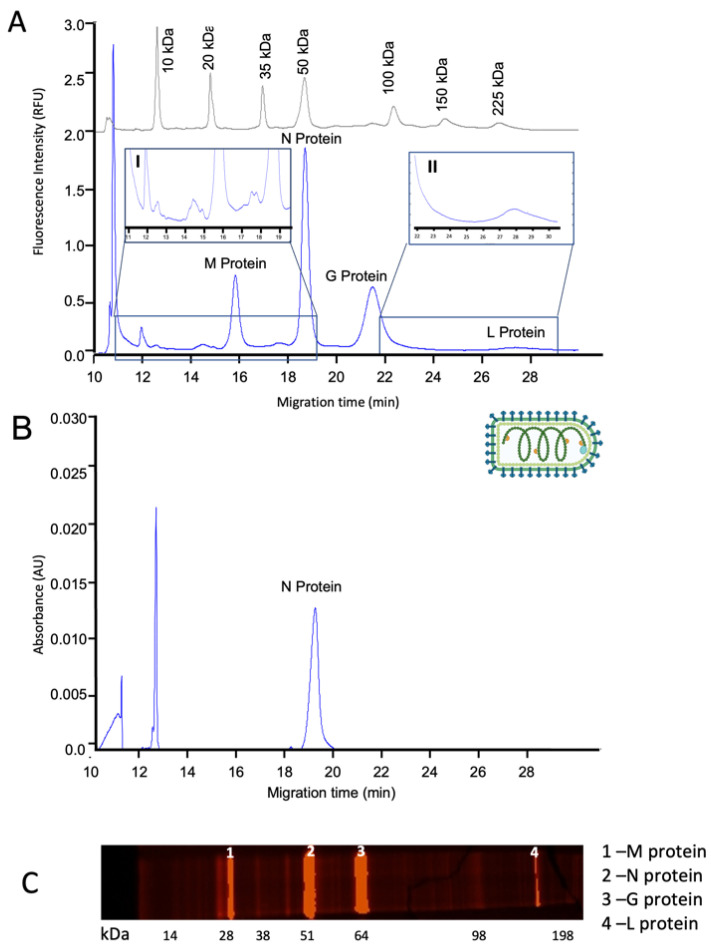
Characterization of MG1 vector. (**A**) Overlay of electropherograms of MW marker (grey line) and purified MG1 sample using CE-SDS with a LIF detector (blue line). I, Zoom-in of impurities profile (RT 11 to 19 min), and II, Zoom-in in L protein elution (RT 22 to 28.5 min). (**B**) Electropherogram of the purified MG1 samples using CE-SDS with a UV detector (**C**) SDS-PAGE with Sypro Ruby staining.

With a similar approach used for rAAV analysis, we used the Maraba MG1 vector as proof of concept to evaluate the capability of CE-SDS LIF methodology for enveloped virus analysis, as these vectors present extra challenges in manufacturing and there is a lack of suitable analytical tools for assessing their quality. MG1 is a vesicular stomatitis virus with a bullet shape, belonging to the *rhabdovirus* family. This oncolytic vector is made up of four proteins, G, M, N, and L, with theoretical molecular weights (MW) of 60, 20–25, 47, and 242 kDa, respectively. Using CE-SDS LIF methodology, we were able to detect the four main proteins of the viral vector with excellent separation at MT 15.76, 18.61, 21.38, and 27.02 min, respectively (Figure 2A). The protocol used in this work for viral proteins’ detection uses a combination of chemical (SDS detergent) and thermal denaturation. This has been proven to work with rAAV particles, which are known to be resistant to several chemical agents, as described by Srivastava et al. [32]. Moreover, we also showed that this denaturation method is highly efficient with enveloped viruses such as lentivirus and Maraba virus, also in combination with CE-SDS LIF analysis offers a good separation of enveloped virus proteins. Potentially, other enveloped viruses described to be resistant to chemical degradation, as SARS-CoV2, could also be successfully analyzed with this approach [33].

**Figure A1:**
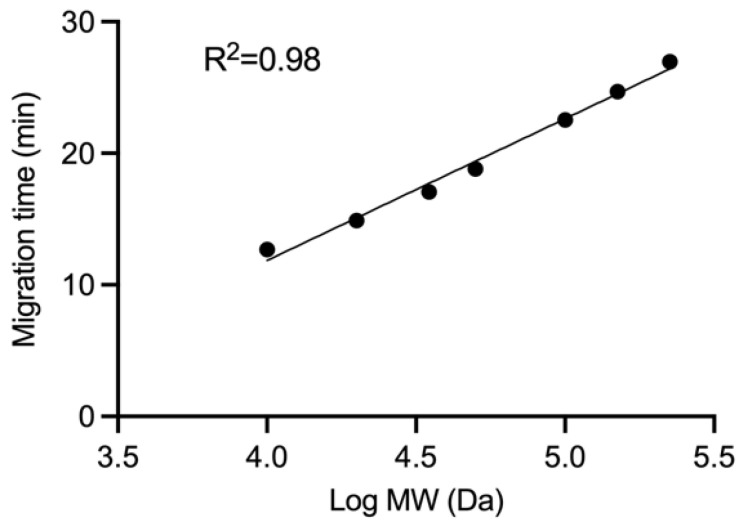
Calibration curve of Migration time (min) vs log MW (Daltons) for molecular weight marker standard.

We determined the MW of the observed peaks by extrapolation of the MT of known MW standards ran under the same conditions (Figure A1). The calculated MWs were consistent with the theoretical MW of M, N, G and L proteins, as depicted in Table A1. Moreover, we were able to detect several process-related impurities peaking from MT 11.89 to 17.07 min. When comparing CE detectors, LIF and UV (Figure 2A and Figure 2B, respectively), we concluded that even if we loaded 100 times more sample, it was only possible to detect protein N with the UV light detector (peak area 10 times less intense than with LIF), not being possible to detect the low MW impurities and M, G, and L proteins. Our results are in agreement with previous reports on the characterization of other viral vectors, showing that CE-SDS LIF methods have higher sensitivity than CE-SDS UV [18].

Additionally, we compared CE-SDS LIF methodology with the traditional SDS-PAGE. We used the Sypro Ruby Pro staining, a ready-to-use and ultrasensitive stain for the detection of proteins. This method is commonly used to assess the purity of therapeutic proteins, including rAAV [34]. As with the CE-SDS LIF approach, we were able to detect the presence of the core viral proteins (M, N, G, and L proteins), as well as low MW protein impurities. However, we loaded up ten times more sample than in the CE-SDS LIF approach, which also takes less analysis time (25 min of separation time vs 35 min SDS-PAGE separation time plus overnight staining) and has higher-throughput capabilities. Importantly, SDS-PAGE with Sypro Ruby staining seems to overestimate purity results when compared with CE-SDS LIF method since the method is less sensitive, showing a false higher level of purity.

**Figure 3:**
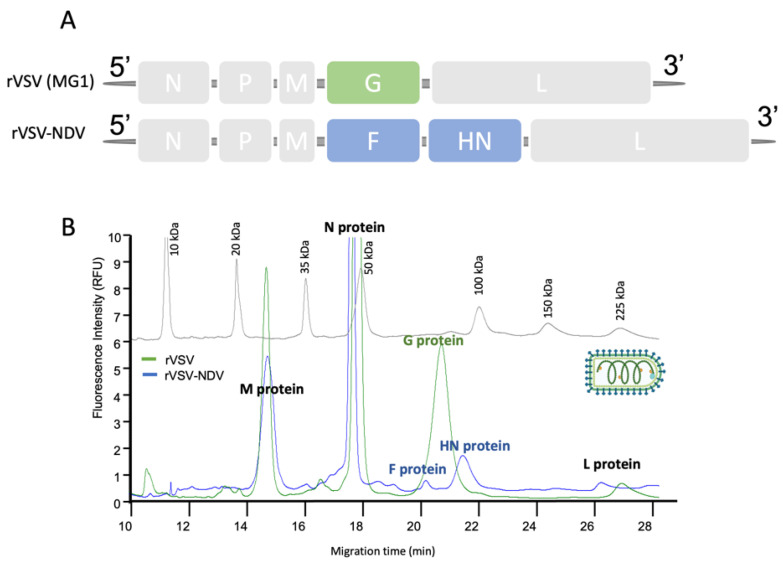
Characterization of chimeric virus rNDV-VSV by CE-SDS LIF. (**A**) Differences in the structural construction of rNDV-VSV and rVSV family (MG1 vector). (**B**) Overlay of the electropherograms of both viral vectors and MW marker.

We also evaluated the versatility of the implemented CE-SDS LIF methodology by comparing different structural constructions of chimeric enveloped viruses. For this, we used a hybrid engineered vector comprising the Newcastle disease virus (NDV) and vesicular Stomatitis Virus (VSV), named rVSV-NDV. This vector includes a conserved VSV backbone, but its glycoprotein has been replaced by the hemagglutinin-neuraminidase (HN) and the modified hyperfusogenic fusion (F) envelope protein of rNDV. This difference, which is depicted in Figure 3A, contributes to the reduction in the cytotoxic effect associated with the rVSV vector in clinical applications [35]. Consequently, while WT viral vector has a G protein with an expected MW of 58 kDa, the chimeric vector does not have G protein and has both F (65 kDa) and HN (75 kDa as a monomer or 150 kDa as a dimer) proteins instead. We explored the possibility to detect these small MW differences by CE-SDS LIF. In fact, this methodology proved to be a valuable technique for the evaluation of this kind of construction alterations. The results obtained for this comparison between the rVSV-NDV vector and rVSV vector are shown in Figure 3B (and Table A1). The calculated MW of the viral proteins are in line with what is described in the literature [36]. Between MT of 20 and 21 min, it is possible to observe a peak corresponding to the MW of protein G in the WT construct (green line) which is not detected in the electropherogram of the rVSV-NDV (blue line). In the latter, we observe the presence of two peaks with a MT of 20.65 and 21.70 min, corresponding to both enveloped viral proteins (F and HN) of rVSV-NDV.

Escandell et al. [14] very recently discussed the need for new analytical tools to replace the classical techniques that do not have high-throughput capabilities, present low sensitivity, resolution, and reproducibility. Considering all these results, we demonstrated that the FQ labelling combined with CE-SDS LIF analysis is a versatile platform for the characterization of enveloped and non-enveloped viruses. The results here obtained for viral vector samples are consistent with other reported labelling approaches used in this CE-SDS platform, such as labelling with the pyrylium dye Chromeo™ P503 [18], in terms of superiority over UV detection and also the classical SDS-PAGE analysis tools. Importantly, we have shown for the first time that the CE-SDS LIF platform is also suitable for the characterization of final samples of enveloped viruses, which extends the described applicability of the method.

### 3.2. Qualitative Analysis of Virus In-Process Purification Samples

**Figure 4:**
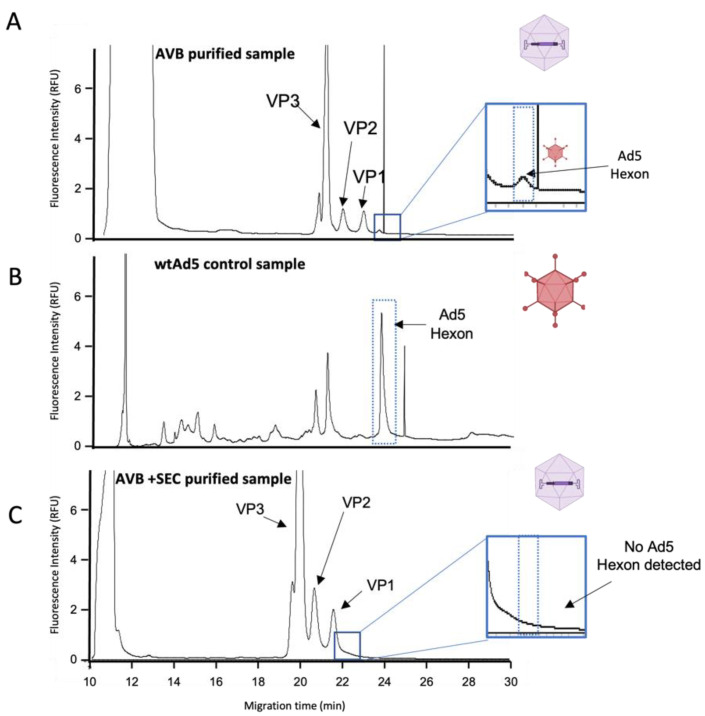
Evaluation of different in-process rAAV samples by CE-SDS LIF. (**A**) Electropherogram of a purified rAAV2 sample with AVB column. (**B**) Electropherogram of wild-type Ad5 control. (**C**) Electropherogram of a purified rAAV2 sample with sequential AVB and SEC columns.

There is a gap in analytical tools for quantitative and qualitative evaluation of virus integrity or/and purification levels during optimization of downstream processing (DSP). There is a clear need for quick, sensitive, and reliable assays to complement the traditional methods used, such as ELISA, qPCR, or SDS-PAGE [5]. To overcome these current limitations in the assessment of DSP, the CE-SDS LIF methodology was explored to characterize in-process samples of the HelaS3 cell line-based rAAV production system. This production system relies on a helper virus infection, usually with wtAd5, which triggers rAAV production. However, to deliver a safe product, the wtAd5 viruses need to be removed during DSP. To attain this, a purification process based on affinity chromatography (AVB Sepharose resin) and size exclusion chromatography (SEC) as a polishing step was employed. Notably, by CE-SDS LIF analysis, we were able to detect traces of wtAd5 contamination in AVB purified sample (Figure 4A), at an MT of approximately 24 min, which corresponds to the migration time of wtAd5 Hexon protein (Figure 4B). The presence of wtAd5 was confirmed also by qPCR of DNAse-treated samples, indicating a wtdAd5 titer of 4 × 10^9^ DNAse Resistant Genomes (DRG)/mL after this first chromatography step. CE-SDS LIF results of the AVB+SEC purified sample showed that SEC removed the wtAd5, as the peak previously observed at an MT of ~24 min was no longer detectable (Figure 4C). These results were further corroborated by qPCR where the wtAd5 titer obtained was 1 × 10^6^ DRG/mL, close to the limit of detection of the assay.

**Figure 5:**
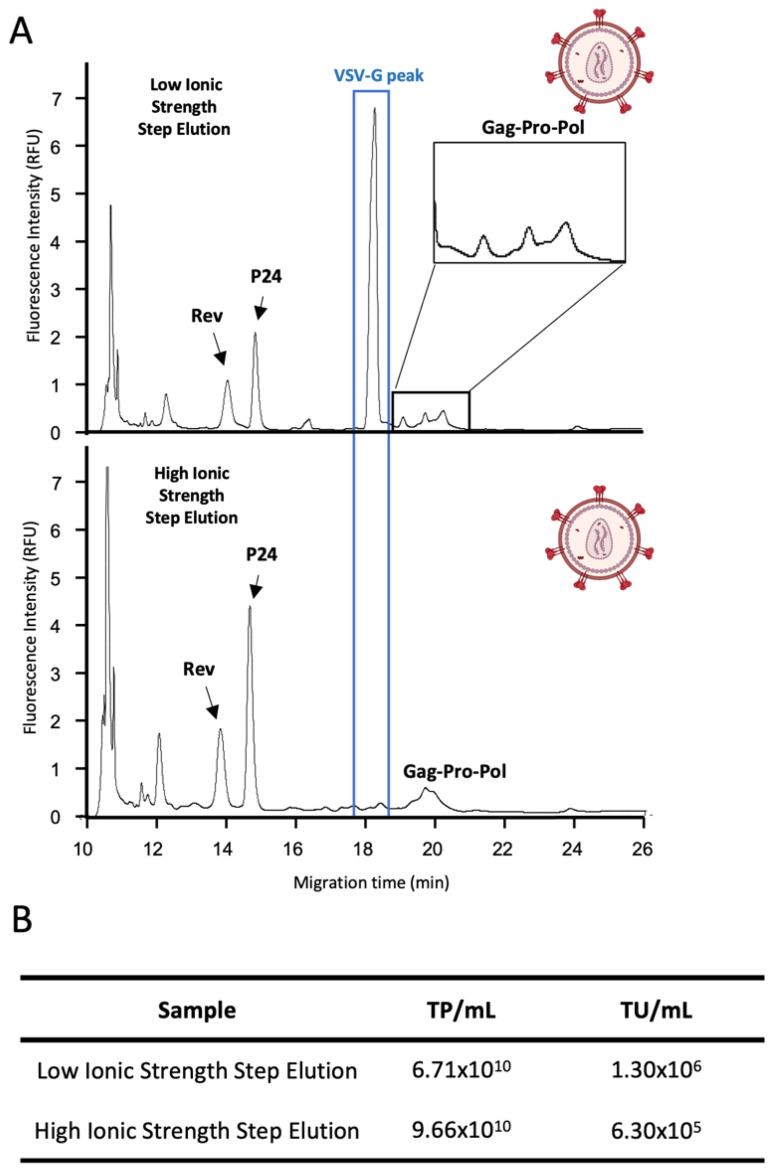
Evaluation of different in-process LV samples by CE. (**A**) Comparison of the two elution fractions of LV IEX chromatographic step (low and high ionic strength, upper and lower panel, respectively). (**B**) Table representation of the TP/mL and TU/mL for both in-processing elution fractions.

LVs are characterized by being fragile and sensitive to DSP, usually due to damage imposed on envelope membrane, which affects their infectivity and functionality. We used the CE-SDS LIF methodology to evaluate LV integrity after ion-exchange membrane (IEX) chromatography, as represented in Figure A3. Two isocratic steps were used for the elution, a lower ionic strength concentration expecting the recovery of the LV (LI sample), and a higher ionic strength concentration to desorb the impurities (HI sample). Figure 5 depicts the electropherograms of both LI and HI samples. CE-SDS LIF analysis of FQ-labelled samples enabled the detection, on the LI sample, of three proteins consistent with the expected LV’s Rev, p24 and Gag-Pol-Pro, at 13.92, 14.71, and 18.80 to 20.12 min, respectively, and the envelope protein VSV-G at 18.15 min (Figure 5A). Regarding Gag-Pro-Pol polyprotein, it is possible to observe three small peaks from 18.80 to 20.12 min approximately (Figure 5A), which are consistent with truncated versions of the polyprotein, as described in the literature [37]. Interestingly, in the case of the HI sample, we were able to detect the proteins Rev, p24, and Gag-Pol-Pro, but not the enveloped VSV-G protein (Figure 5A), suggesting that this elution fraction is composed of defective viruses or other product-related impurities such as non-infectious extracellular vesicles that share common protein with LV [38]. In this work, an ELISA measuring structural p24 protein was used to titrate the samples regarding total particles. The LI and HI samples presented a recovery of 6.71 × 10^10^ TP/mL and 9.66 × 10^10^ TP/mL, respectively. However, when we measured infectious particles by flow cytometry, the HI sample showed a concentration of 6.30 × 10^5^ transducing units (TU)/mL, by comparison with the 1.30 × 10^6^ TU/mL of the LI sample. A possible hypothesis for the discrepancy between ELISA and infectivity assay results is the presence of product-related impurities such as defective LV particles that have lost their enveloped protein (VSV-G) or extracellular vesicles comprising some of the LV proteins in their structure, that could be still detected by p24 ELISA. This could be particularly true in the HI sample since we could not detect the peak for VSV-G protein by CE-SDS LIF (Figure 5A) explaining the lower infectious titer obtained when compared to the LI sample. In fact, in LV manufacturing, the analysis of infectious titer during bioprocessing is of major importance and the most challenging method to be performed. Current established methods, such as flow cytometry and qPCR lack the capability of enabling high throughput sample processing since they require a considerable amount of manual handling [39]. The results herein presented corroborate the need for new reliable analytical tools capable of being integrated with virus-based manufacturing, and this could be partially overcome by the CE-SDS LIF method here described.

Overall, the applicability of the reported CE-SDS LIF methodology was successfully demonstrated for the in-process monitoring of non- and enveloped viruses, making this methodology a suitable analytical platform to be applied during viral biotherapeutics manufacturing.

### 3.3. Method Qualification for Virus-Based Targets Quantification

**Figure 6:**
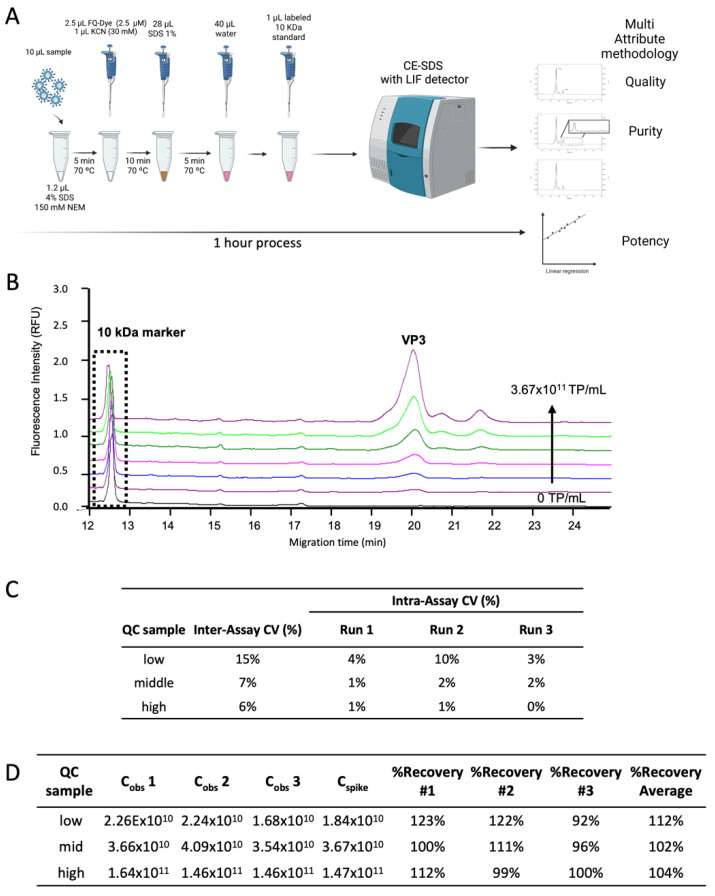
Applicability of the CE-SDS LIF as a quantification method for viral-based manufacturing. (**A**) Schematic representation of the workflow performed for quantification approach. (**B**) Overlay of the electropherograms of the standard calibration curve from 0 to 3.67 × 10^11^ TP/mL. (**C**) Intermediate precision determination, comparison between the calculated quality control (QC) samples concentration with the theoretical concentration (1.84 × 10^10^, 3.67 × 10^10^, 1.47 × 10^11^ TP/mL). (**D**) Accuracy results for the 3 QC samples of reference standard AAV2: low, mid, and high range. The recovery (%) was calculated by the C_obs_ to C_spike_ ratio for each run multiplied by 100. C_obs_—extrapolated concentration of QC samples from the calibration curve; C_spike_—theoretical concentration of QC samples.

ELISA is the gold standard method for rAAV capsid/viral particle quantification [40]. However, as discussed above, this method presents several limitations: it is time-consuming, has high inter-assay variability, and is serotype dependent (in the case of rAAV). New methods for capsid quantification are being developed, such as SEC with Multi-Angle Light Scattering detector (SEC-MALS) or Biolayer Interferometry (BLI). However, SEC-MALS lacks sensitivity and BLI requires serotype-specific antibodies [41]. Given the need for accurate, sensitive, and serotype-independent quantification, a CE-SDS LIF rAAV quantification method was qualified (following the ICH guidelines for method qualification [41]). The workflow used is depicted in Figure 6A.

In this work, the specificity, precision, accuracy, linearity, LOD, and LOQ of the method were assessed to demonstrate that the method is suitable for the specified purpose.

**Figure A2:**
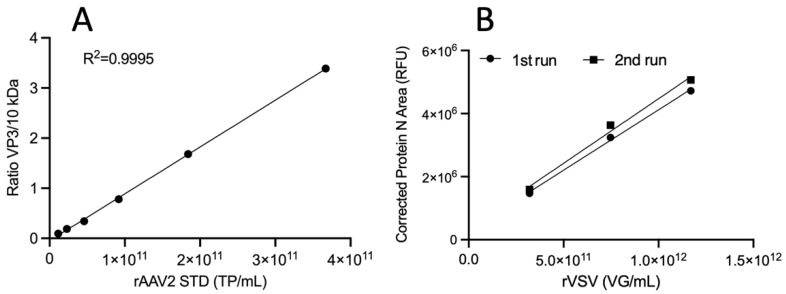
(**A**) Linear representation of fluorescence response vs rAAV2 total particles (TP/mL) concentration (average of analyst 1 run 1 assay). (**B**) Linear representation of fluorescence response vs MG1 concentration (VG/mL).

**Figure A3:**
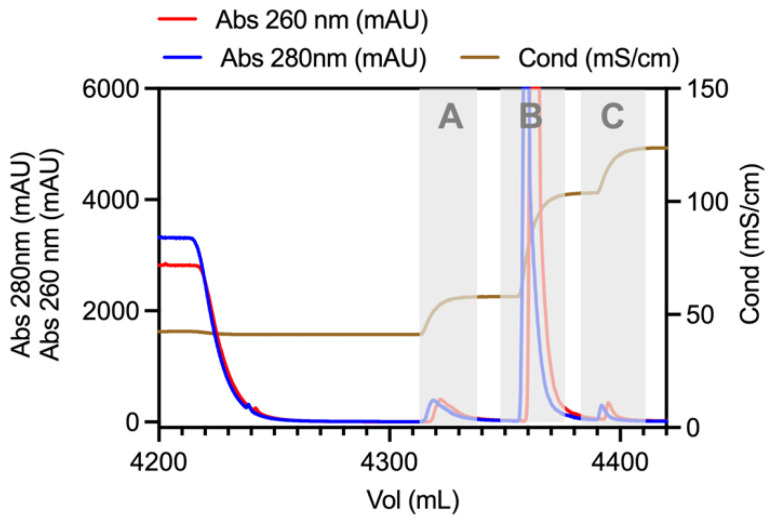
Representative chromatogram of LV purification using anion exchange chromatography (AEX) with step elution. (A) Washing step. (B) Low Ionic Strength Step Elution. (C) High Ionic Strength Step Elution.

The specificity was proven by the absence of peaks consistent with the viral protein of interest (VP3) in the electropherogram of the formulation buffer (blank). Evaluation of method linearity was based on the LIF detector response when injecting six concentration levels of rAAV2 reference material (1.15 × 10^10^, 2.30 × 10^10^, 4.59 × 10^10^, 9.18 × 10^10^, 1.84 × 10^11^, and 3.67 × 10^11^ TP/mL) with a constant concentration of the internal control (10 kDa marker). This spike with an internal control was used to normalize the peak areas between runs, ensuring that the measurements were not affected by fluorescence decay over time. The electropherograms for the six concentration levels of rAAV2, are depicted in Figure 6B. A linear response was obtained by plotting the VP3/10 kDa marker peak area ratio versus the corresponding six concentration levels of rAAV2 used (Figure A2A). In the concentration range evaluated, it was possible to obtain a correlation with R^2^ higher than 0.996, considering all nine standard curves assessed in three experiments (three independent sequences with triplicates each).

The intermediate precision was also assessed along with three independent sample preparations and measurements. The measurements were performed by two different analysts (analyst 1, run 1 and run 2; and analyst 2, run 3) on three different days, representing three assays in total. Each assay included three replicates of the standard reference material curve and the three-quality control (QC) samples. These comprised high (1.53 × 10^11^ TP/mL), medium (3.83 × 10^10^ TP/mL), and low (1.43 × 10^10^ TP/mL) concentrations, within the standard curve range. CE-SDS LIF results showed good intermediate precision with CV values between experiments (inter-assay) equal to or lower than 15%, with the highest values corresponding to the low range QC sample (Figure 6C). Moreover, intra-assay CV results were very consistent and lower than 10%, revealing good repeatability.

The accuracy was assessed over the three concentration levels (QC samples) in triplicate, by calculating the percent recovery (ratio between the extrapolated concentration from the calibration curve and the theoretical concentration of each QC sample). The recovery average obtained (Figure 6D) for the QC samples was 112%, 102%, and 104% for QC low, mid, and high range, respectively. Notably, this value is within the acceptance criteria of FDA guidelines [42], except for the low range QC in two of the assays, showing values of 122 and 123%. Thus, for lower concentrations the accuracy may decrease. Nevertheless, the overall accuracy of the three QC samples was within this range, confirming the suitability of the method for quantification of AAV2 in the conditions herein used.

All these data showed the linearity, precision, accuracy, and specificity of the CE-SDS LIF method under the conditions described. Moreover, the results obtained were in line with the criteria considered acceptable by the regulatory authorities for method validation. The LOD and the LOQ were calculated based on signal-to-noise ratio (described in [41]) for all three assays, considering both analysts (Table A2). The average estimation for LOD and LOQ was 3.19 × 10^9^ and 9.68 × 10^9^ TP/mL, respectively. These results also demonstrate the high sensitivity and capability of this method to detect and determine low-range concentrations of rAAV samples. A desalting step could be performed to increase the sensitivity of the quantification method, as shown in Section 3.1. Although this may reduce precision and accuracy (due to material losses), it decreases the variability associated with the sample matrix interferences (buffer exchange). Nevertheless, the method’s LOQ, determined in this workflow, is suitable for rAAV-based pharmaceuticals, where the dosage in the final product could be from 3 to 5 orders of magnitude higher [14]. When compared to orthogonal methods which measure capsid titer, the CE-SDS LIF qualified method showed 2–3 orders of magnitude greater sensitivity than serotype independent methods, such as SEC-MALS or Optical density, presenting equivalent sensitivity to serotype dependent methods [41].

This workflow herein proposed is described as a high-throughput method since it enables the analysis of several samples in relatively short period of time. As showed in Figure 6 sample preparation and analysis could be achieved in approximately 1 h. This time is mostly influenced by the several incubation times needed (denaturing and labeling) combined with the separation of proteins in CE-SDS (25 to 35 min). Furthermore, operator dependence factor could be greatly diminished with automation systems for sample preparation. This demonstrates its applicability as a high-throughput platform for the investigation of various rAAV products and serotypes and with little operator-dependence. Importantly, the electropherograms obtained could also be used to measure purity and VP3/VP1 ratio with high repeatability and sensitivity, all defined as CQAs for rAAV-based biopharmaceuticals.

After qualifying the method for rAAV quantification, we evaluated its applicability for enveloped viral targets. Three MG1 samples from different time points in the DSP train were analyzed. Using the above described AAVs concentrations of 3.20 × 10^11^, 7.47 × 10^11^, and 1.17 × 10^12^ VG/mL were extrapolated. The corrected protein N peak area, as shown in Figure A2B, revealed good linearity of fluorescence response vs sample concentration, with R^2^ higher than 0.990 (*n* = 2, independent runs). This indicates the potential of the method for the quantification of more complex particles, such as enveloped-based targets. However, to improve the quantification method accuracy the use of a specific standard sample is required. However, there is not any commercially available reference standard for this virus in Europe, which did not allow us to further explore the quantification method for MG1.

## 4. Conclusions

Virus-based pharmaceuticals play a critical role as emerging tools for cell and gene therapy applications, and they require an extensive characterization to guarantee the safety and efficacy of the therapies. This work describes the implementation of an FQ labelling procedure coupled to a CE-SDS-LIF detector, making this platform highly sensitive and suitable for in process and final product virus-based samples. Besides this, it was described for the first time that a CE-SDS LIF method is suitable for the qualitative analysis of in-process samples and final product of enveloped virus-based targets, such as lentiviral vectors or Maraba MG1, overcoming current needs of the field. Additionally, the CE-SDS LIF was also explored to implement a quantification method for purified rAAV, following the ICH guidelines for method qualification. This could contribute as a step forward to have this method used under GMP conditions.

Overall, our results showed that CE-SDS LIF has the potential to become a standard approach for the analysis of virus-based products in a near future, owing to its superior performance and compatibility with GMP compliance.

## Figures and Tables

**Table 1:** Description of the experimental conditions used for conditioning and shutdown methods.

Solution	Aim	Operation Conditions (Conditioning)	Operation Conditions (Shutdown)
**0.1 M NaOH**	cleaning	10 min, 20 psi, forward	10 min, 70 psi, forward
**0.1 M HCl**	neutralization	5 min, 20 psi, forward	5 min, 50 psi, forward
**LC-MS grade water**	removal of acid residues	2 min, 20 psi, forward	2 min, 50 psi, forward
**SDS-MW gel buffer**	filling	10 min, 70 psi, forward	10 min, 70 psi, forward
**SDS-MW gel buffer**	equilibration	10 min, 15 kV, forward	10 min, 15 kV, reverse polarity

## Data Availability

Not applicable.

## Appendix A

**Table A1:** Molecular weight (MW) determination for the different viral proteins of MG1 and rVSV-VSV obtained by extrapolation of migration time from the calibration curve.

	Protein	MT (min)	log MW (Da)	MW (kDa)
MG1	M	15.76	4.36	23
	N	18.61	4.63	42
	G	21.38	4.88	77
	L	27.02	5.41	256
rVSV-NDV	M	15.66	4.35	23
	N	18.55	4.62	42
	F	20.65	4.82	66
	HN	21.70	4.91	82
	L	27.02	5.41	256

**Table A2:** CE-SDS LIF method qualification for quantification of AAV using a Reference standard AAV2 sample; LODs and LOQs and their average estimation are shown. LODs and LOQs were calculated based on signal to noise ratio methodology for each experiment.

		Replicates			
		1	2	3	Average
Analyst 1 Run1	LOD (TP/mL)	2.86 × 10^9^	3.10 × 10^9^	2.31 × 10^9^	2.76 × 10^9^
	LOQ (TP/mL)	8.66 × 10^9^	9.40 × 10^9^	6.99 × 10^9^	8.35 × 10^9^
Analyst 1 Run2	LOD (TP/mL)	3.41 × 10^9^	6.08 × 10^9^	4.49 × 10^9^	4.66 × 10^9^
	LOQ (TP/mL)	1.03 × 10^10^	1.84 × 10^10^	1.36 × 10^10^	1.41 × 10^10^
Analyst 2 Run3	LOD (TP/mL)	1.98 × 10^9^	2.80 × 10^9^	1.71 × 10^9^	2.17 × 10^9^
	LOQ (TP/mL)	6.01 × 10^9^	8.50 × 10^9^	5.18 × 10^9^	6.56 × 10^9^
